# Advancing the use of Long‐Acting Extended Delivery formulations for HIV prevention in sub‐Saharan Africa: challenges, opportunities, and recommendations

**DOI:** 10.1002/jia2.26115

**Published:** 2023-07-13

**Authors:** Nyaradzo M. Mgodi, Grant Murewanhema, Enos Moyo, Chesterfield Samba, Godfrey Musuka, Tafadzwa Dzinamarira, Joelle M. Brown

**Affiliations:** ^1^ University of Zimbabwe Clinical Trials Research Centre Harare Zimbabwe; ^2^ Unit of Obstetrics and Gynecology Faculty of Medicine and Health Sciences University of Zimbabwe Harare Zimbabwe; ^3^ Medical Centre Oshakati Namibia; ^4^ GALZ Harare Zimbabwe; ^5^ International Initiative for Impact Evaluation Harare Zimbabwe; ^6^ School of Health Systems and Public Health University of Pretoria Pretoria 0084 South Africa; ^7^ Department of Epidemiology & Biostatistics University of California San Francisco School of Medicine San Francisco California 94158 USA

**Keywords:** adolescent girls and young women, Africa, men who have sex with men, PrEP, prevention, stigma, long‐acting

## Abstract

**Introduction:**

The burden of HIV in sub‐Saharan Africa (SSA) remains unacceptably high, and disproportionately affects girls and women. While the introduction of oral HIV pre‐exposure prophylaxis (PrEP) in 2012 revolutionized HIV prevention, its effectiveness is dependent on user adherence and its implementation in SSA has faced numerous challenges. Patient‐level, interpersonal and structural barriers, including, for example, daily pill burden, side effects, lack of partner support, testing and disclosure, and costs have been found to reduce adherence to oral PrEP.

**Discussion:**

Long‐acting extended delivery (LAED) formulations for PrEP, such as injectable long‐acting cabotegravir (CAB‐LA) and dapivirine vaginal ring (DPV‐VR) are critical additions to the HIV prevention toolkit and are especially important for populations such as adolescent girls and young women (AGYW) and other key populations who remain at significant risk of HIV acquisition while facing substantial barriers to preventive services. These LAED formulations have been shown to result in better adherence and fewer side effects, with CAB‐LA being superior to oral PrEP in reducing the risk of HIV acquisition. They can be used to overcome user burden and adherence challenges. However, the successful rollout of the DPV‐VR and CAB‐LA may be hampered by issues such as a shortage of healthcare providers (HCPs), inadequate parenteral medication infrastructure, increased workload for HCPs, patient concerns, the price of the medications and the possibility of drug resistance.

**Conclusions:**

SSA must develop laboratory capabilities for monitoring patients on LAED formulations and enhance research on developing more non‐injectable LAED formulations. There is a need to train and retain more HCPs, implement task shifting, invest in healthcare infrastructure and integrate healthcare services. To reduce costs and improve availability, the region must advocate for patent license waivers for LAED formulations and procure drugs collectively as a region.

## 1 INTRODUCTION

### 1.1 The burden of HIV in sub‐Saharan Africa

Despite major advances in HIV prevention and treatment, the global burden of HIV remains unacceptably high. Approximately 38 million people are currently living with HIV. In 2021 alone, 650,000 people died from AIDS‐related illnesses and there were 1.5 million new HIV acquisitions, representing a marginal decline of 31% since 2010 [1, 2], far below the target of a 75% reduction in new acquisitions for 2020 set by the United Nations General Assembly in 2016 [1, 2].

HIV/AIDS disproportionately affects sub‐Saharan Africa (SSA). In 2021, 65% of all HIV‐related deaths and 58% of all new HIV acquisitions occurred in SSA. Heterosexual women and girls in the region are especially burdened by HIV, accounting for 59% of all HIV acquisitions in SSA [1, 2]. This gender disparity starts at a young age with adolescent girls and young women (AGYW) aged 15–24 accounting for 25% of all new HIV acquisitions in the region despite representing just 10% of the population. Key populations (KPs) and their sex partners, including sex workers, men who have sex with men (MSM), people who inject drugs, and transgender people account for about half of new HIV acquisitions in SSA [1].

### 1.2 Current progress with oral pre‐exposure prophylaxis rollout

A decade after its approval for use among people at substantial risk of HIV acquisition, oral pre‐exposure prophylaxis (PrEP) access is still concentrated in a relatively small number of countries [3], and PrEP distribution within countries is not equal, resulting in a significant unmet need for PrEP [4]. To date, nearly 3.9 million people have initiated PrEP globally. While impressive, this is thought to represent only 10% of those who are at risk and could benefit from PrEP [3].

### 1.3 Barriers to oral PrEP use in SSA

Underlying multi‐level barriers have slowed the progress of PrEP implementation in SSA. Lack of adherence to oral PrEP has been associated with daily pill burden, side effects, stigma, fear of partner violence, religious beliefs and costs [5, 6, 7]. Long waiting times at health centres, dissatisfaction with care and reduced privacy due to inadequate consulting rooms are among the factors in the health system that hinder adherence [8]. Other health system factors that reduce adherence to PrEP include existing delivery practices requiring frequent healthcare facility visits for medication refills and negative attitudes by healthcare providers [9]. The uptake of healthcare services in general, and PrEP in particular, is lower among KPs in SSA due to widespread stigma, prohibitive legislation and discrimination [7]. In 2017, South Africa reported an oral PrEP uptake of 12% among sex workers and 43% among MSM [10]. Community‐based distribution of oral PrEP is acceptable and may overcome barriers associated with clinic‐based PrEP distribution and achieve high PrEP uptake and retention [11].

### 1.4 The need for long‐acting extended delivery formulations for HIV prevention

These challenges and unrelenting incident HIV acquisitions highlight the urgent need for a wider PrEP toolkit that includes long‐acting extended delivery (LAED) PrEP formulations with more convenient dosing schedules [12]. However, only some of the PrEP delivery challenges will be addressed by these LAED formulations. In this article, we provide an overview of injectable long‐acting cabotegravir (CAB‐LA) and dapivirine vaginal ring (DPV‐VR), discuss the possible implementation challenges and opportunities that SSA countries will likely face during the rollout of LAED PrEP and suggest recommendations to address the challenges.

## 2 DISCUSSION

### 2.1 Available LAED formulations

Table 1 summarizes the salient features of CAB‐LA and DPV‐VR. Cabotegravir is a second‐generation integrase strand transfer inhibitor (INSTI) with a high barrier to resistance [20]. The HPTN‐083 and 084 studies demonstrated that injectable CAB‐LA was safe and superior to daily oral tenofovir/emtricitabine for HIV prevention across two cohorts at increased risk of HIV acquisition through vaginal and anal intercourse; cisgender women and cisgender men/transgender women who have sex with men, respectively [14, 21]. Compared to participants on oral PrEP, participants receiving intramuscular CAB‐LA 600 mg once every 2 months had a 66% and 89% reduction in HIV acquisition in the two studies. CAB‐LA was well tolerated and was demonstrated to have acceptable pharmacokinetic and safety profiles. Most adverse events were mild or moderate and balanced between the two study arms [13]. Injection site pain (ISP), headache, diarrhoea, nausea and exhaustion are a few self‐limiting side effects of CAB‐LA. ISP became less frequent with subsequent CAB‐LA doses. There was high adherence to CAB‐LA across the efficacy studies [13, 21].

**Table 1:** Injectable CAB‐LA and dapivirine vaginal ring for HIV prevention

Drug for HIV prevention	References	Type	Approval	Effectiveness	Side effects/safety	Route/dosing	Resistance	Other considerations
Long‐acting injectable cabotegravir (CAB‐LA) nanosuspension	[13–16]	Second‐generation INSTI	Currently approved by WHO and FDA	More effective than oral PrEP with tenofovir/emtricitabine: 66%–89% reduction in HIV acquisition versus oral tenofovir/emtricitabine	Side effects include pain at the injection site, headaches, diarrhoea, nausea and exhaustion. Acceptable pharmacokinetic and safety profile	Route: intramuscular injection Dosing: every 4 weeks for 2 months, then every 8 weeks	Prolonged half‐life Stable in the presence of frequent INSTI mutations	Cost‐effective in some settings High acceptability among patients in SSA
Long‐acting dapivirine vaginal ring	[17–19]	NNRTI	Licenced by FDA and WHO. Approved for use in Kenya, Malawi, Uganda, Rwanda, South Africa and Zimbabwe	29% HIV risk reduction versus placebo ring	Strong safety profile	Route: intravaginal insertion Dosing: monthly	No related resistance	Acceptable in SSA Potential to be combined with levonorgestrel for a single multipurpose ring. Stored at room temperature (no cold chain); 5‐year shelf‐life

Abbreviations: INSTI, integrase strand transfer inhibitor; WHO, World health organization; FDA, U.S. Food and drug administration; SSA, sub‐Saharan Africa.

Pregnancy incidence in HPTN‐084 was 1.5 per 100 person‐years in the CAB‐LA group, with no congenital abnormalities reported. HPTN‐083 data suggest that gender‐affirming hormone therapy does not affect cabotegravir pharmacokinetics [13]; the World Health Organization (WHO) considers CAB‐LA a safe and effective HIV prevention strategy for transgender women [15]. Data from these studies supported United States Food and Drug Regulatory Agency (FDA) approval and WHO recommendation for CAB‐LA as PrEP, with subsequent registrations filed in the United States, Australia, Botswana, Brazil, Kenya, Malawi, South Africa, Uganda and Zimbabwe.

CAB‐LA 600 mg is administered 4 weeks apart for the first two doses and every 8 weeks after that for PrEP [9]. The prolonged half‐life of CAB‐LA allows for a delay in follow‐up dose of up to 8 weeks without dose alteration; however, if the follow‐up injection is missed by more than 8 weeks or more, a higher loading dose will be required before returning to routine [22]. CAB‐LA should not be co‐administered with potent liver enzyme inducers like rifampicin and carbamazepine, which decrease its serum concentration by almost 60% [14]. CAB‐LA is cost‐effective when delivered with other preventive measures or given priority among certain populations, particularly women [15]. Participants in clinical trials reported overall satisfaction with CAB‐LA [13]. More than 75% of participants in a study of women from the United States and Africa regarded long‐acting injectables as highly satisfactory. The study concluded that women's demand for a long‐acting injectable PrEP might be more significant in Africa than in US settings [16].

The DVR‐VR is formulated as a flexible silicone ring that slowly releases the non‐nucleoside reverse transcriptase inhibitor (NNRTI) dapivirine. The monthly vaginal ring is acceptable for use in SSA [17] and has the potential for better adherence than oral PrEP. It is a discreet, woman‐initiated, easy‐to‐use intervention. To date, no related resistance has been reported. In July 2020, the European Medicines Agency announced a positive opinion about the ring for use by women 18 years and older to reduce their HIV risk [12]. The opinion was based on data from two large Phase III randomized trials which involved approximately 4,600 women in Malawi, Uganda, South Africa and Zimbabwe. Both studies demonstrated that the ring has a strong safety profile and reduced the risk of HIV acquisition by approximately 30%. Subsequent subgroup analyses among ASPIRE trial women who had high ring adherence according to residual drug levels in the DPV‐VR suggested a greater HIV risk reduction of 50% or higher [18, 19].

The ring is now approved by several SSA countries. Though less efficacious than oral or injectable PrEP, it may be an essential risk‐reduction tool for women unable, or unwilling to take oral or injectable PrEP. The addition of the ring to the prevention toolbox strengthens the development of next‐generation antiretroviral‐based vaginal rings/films with the potential to be combined with levonorgestrel as a single multipurpose prevention technology [23].

### 2.2 Challenges, opportunities and recommendations in the implementation

SSA will face several challenges in rolling out LAED formulations. Below, we discuss challenges related to infrastructure, trained personnel, product‐related fears, HIV‐related stigma, costs, drug resistance, guidelines and research, and then discuss opportunities and recommendations in the implementation of LAED in SSA.

#### 2.2.1 Infrastructure

Administration of injectable formulations, such as CAB‐LA, requires adequate rooms for client confidentiality, needle supply and suitable disposal facilities for used needles. Some facilities may lack adequate space and the infrastructure to properly dispose of the increased volumes of used needles, increasing the risk of transmission of blood‐borne infections [24]. To address the additional infrastructure needs, countries may need to integrate HIV services with other healthcare services, such as sexual and reproductive health services, antenatal/postnatal clinics and men's clinics [25]. A study conducted in Malawi to assess the integration of reproductive health services into HIV care revealed that such integration required minimal additional resources [26].

#### 2.2.2 Trained personnel

SSA has the world's lowest healthcare provider (HCP) to population ratio [25]. The need for intramuscular administration of long‐acting injectables presents a problem in the region because registered nurses and doctors are required to administer the injections. These HCPs authorized to deliver parenteral medications are not present in adequate numbers across healthcare facilities in the region [25]. We recommend that SSA countries amend their policies to permit other HCPs, such as enrolled nurses and primary care nurses, to administer parenteral medications after proper training [25]. Additionally, we recommend that CAB‐LA preparation be done at the point of injection just like other injectable medications like depot medroxyprogesterone acetate (DMPA), with special attention to body mass index calculation for needle size determination before administration.

A challenge that can arise when rolling out new interventions is an increased workload for the few available HCPs. Before delivering CAB‐LA, clients must first be evaluated to determine their eligibility. Additionally, patients will require observation for at least 10 minutes following the injection [27]. Although community‐based oral PrEP delivery models have increased the uptake of oral PrEP, the same models may be difficult to use in the administration of injectable LAED formulations. De‐medicalizing the rollout of LAED formulations and appropriate task shifting may help to address this challenge. For example, community health workers could be trained on the safe administration of injections, and strategies devised for the safe disposal of the sharp materials used. While it is not possible to make use of community‐based adherence groups that take turns going to the clinic or community product pick‐up sites to collect the drugs for the whole group when using injectable LAED formulations, this approach may be feasible for DPV‐VR.

HCP attitude and wellbeing are important attributes in health provision [8]. Countries in SSA should improve working conditions for HCPs and invest in the training of more HCPs to ensure that healthcare facilities are adequately staffed. This will improve staff morale and reduce the workload, ensuring that client waiting times are not long and resulting in improved client satisfaction. Satisfied patients are more likely to be adherent and have favourable management outcomes.

#### 2.2.3 Product‐related fears and concerns

Some patients fear needles or are scared of ISP, hence might not be willing to switch from oral PrEP to injectable PrEP. Given the time it takes before the next injection, some patients might worry about insufficient protection. Furthermore, it could still be challenging to visit the medical facilities every 2 months for CAB‐LA [15]; though there is potential for administration every 12 weeks, a schedule which would coincide with DMPA injection for contraception. In addition, patients should be well informed about the side effects of the long‐acting formulations to improve compliance and reduce the discontinuation of the drugs without consulting HCPs [28].

#### 2.2.4 HIV‐related stigma

HIV‐related stigma can prevent patients from accessing services [29]. To reduce stigma and discrimination, communities should be engaged in formulating HIV programmes. This will make clients feel they are part of the programmes and improve implementation success [25]. Well‐informed LAED messaging should be used to allay myths and misconceptions to reduce stigma and discrimination against those using them and improve uptake [25].

#### 2.2.5 Costs

Most SSA countries may not be able to afford CAB‐LA and DPV‐VR at the indicated cost of USD18/dose and USD6/ring, respectively, even though they may be considered cost‐effective in some settings [25]. CAB‐LA patent expires in 2031; if the manufacturers of long‐acting formulations do not waive their patent licenses, it will take a long time for countries in SSA to be able to manufacture affordable generics [30]. Countries in SSA should, therefore, advocate for technology transfer and for drug developers to expeditiously waive their patent licenses so that cheaper generic forms can be produced regionally, ensuring that those at the greatest risk of HIV acquisition have access to PrEP. Furthermore, countries in SSA must procure the medications as a region to negotiate for more significant discounts with manufacturers.

#### 2.2.6 Drug resistance

CAB‐LA has a half‐life of 20–65 days and has been detected in some patients’ blood up to 1 year after the last injection. However, the concentrations detected were suboptimal, which may lead to drug resistance [22]. Without robust follow‐up of patients lost to follow‐up, drug resistance may increase, driving HIV care costs up. Mathematical modelling shows that AIDS deaths are predicted to decline in 99% of scenarios where CAB‐LA is implemented. However, INSTI resistance is of concern in SSA, where most first‐line regimens include dolutegravir. Resistance may occur when CAB‐LA is initiated in a recently infected individual with infection not yet detected by third‐generation antibody tests used as the standard of care in SSA or as a breakthrough infection during CAB‐LA use [31, 32]. To address this challenge, countries in SSA should build the capacity to monitor patients on long‐acting injectable formulations for HIV transmission and drug resistance and invest in robust HIV testing algorithms that can detect incident HIV infections early.

#### 2.2.7 Gaps in guidelines and research

Several countries in SSA have now incorporated the DPV‐VR in national PrEP guidelines. The CATALYST study will spearhead the two‐stage implementation of an enhanced service delivery package providing a choice of oral PrEP, DPV‐VR and CAB‐LA among women at U.S. President's Emergency Plan for AIDS Relief (PEPFAR) delivery sites in several African countries [33]. The exclusion of DPV‐VR from the Essential Medicines List for PEPFAR will likely delay widespread availability to beyond 2026 unless the Global Fund allows earlier procurement of the ring. The current lack of national guidance for CAB‐LA means that healthcare facilities in SSA will take longer to embrace it and rollout may be further delayed. Guidelines for the rollout of LAED PrEP formulations are urgently needed and must be human rights‐based, with guidance on appropriate task‐shifting, and must normalize the use of the new products so those who need PrEP most can access it.

Data on the safety and efficacy of both CAB‐LA and DPV‐VR are not yet widely available among people who inject drugs, sex workers, pregnant and breastfeeding persons, gender‐diverse populations and young people under 18 years. Implementation studies to address these gaps are planned for the Americas, Asia and SSA. SSA will also need to proactively engage KPs in the rollout of these new interventions to pre‐empt socio‐cultural barriers and gender inequalities that currently limit access to existing oral PrEP and other sexual reproductive health (SRH) services.

Currently, several novel, sustained‐release next‐generation drug delivery systems, such as implantable technologies and injectable depo systems, are at various stages of the research and development pipeline [12]. If these alternative vehicles of drug delivery are found safe and effective, they will reduce the frequency of administration, and potentially reduce the number of HCPs required for HIV care and the workload among HCPs [24].

## 3 CONCLUSIONS

Long‐acting formulations of PrEP, such as CAB‐LA and DPV‐VR, offer several advantages over daily oral PrEP. Nevertheless, the rollout of these long‐acting formulations in SSA may face challenges, such as a shortage of HCPs, inadequate infrastructure for parenteral medication, increased workload for HCPs, patients’ concerns, drug cost, stigma, drug resistance, and gaps in guidelines and research. To address these implementation challenges, we recommend that the region utilizes a multi‐sectoral approach to build laboratory capacity for monitoring patients on long‐acting formulations, and enhance research on the development of other controlled release technologies that are accepted by end‐users and meet the needs of the community. SSA should provide a human‐rights‐based delivery service, train and retain more HCPs, invest in healthcare infrastructure, integrate health services, advocate for waiving patent licenses for long‐acting injectables and procure drugs together as a region. We anticipate that this will accelerate efforts to expeditiously deliver CAB‐LA and DPV‐VR as part of a comprehensive HIV prevention package to persons who need it most, with the ultimate aim of improving the quality of life for at‐risk populations in SSA.

## COMPETING INTERESTS

The authors declare no competing interests.

## AUTHORS’ CONTRIBUTIONS

NMM—Conceptualization; Supervision; Writing original draft; GM—Writing original article; EM and CS–Writing original draft; GM, TD and JMB—Writing review and editing.

## FUNDING

GM was supported by the Office of Research on Women's Health and the Fogarty International Center of the National Institutes of Health under Award Number D43TW009343 and the University of California Global Health Institute.
